# Choosing the right timing for interval debulking surgery and perioperative chemotherapy may improve the prognosis of advanced epithelial ovarian cancer: a retrospective study

**DOI:** 10.1186/s13048-021-00801-4

**Published:** 2021-03-27

**Authors:** Dengfeng Wang, Guonan Zhang, Chunrong Peng, Yu Shi, Xunwei Shi

**Affiliations:** Gynecologic Oncology Center, Sichuan Cancer Hospital & Institute, Sichuan Cancer Center, School of Medicine, University of Electronic Science and Technology of China, No. 55, Section 4, South Renmin Road, Chengdu, 610041 China

**Keywords:** Ovarian cancer, Neoadjuvant chemotherapy, Surgery, CA125, Prognosis

## Abstract

**Background:**

Primary debulking surgery (PDS) is the main treatment for patients with advanced ovarian cancer, and neoadjuvant chemotherapy (NACT) is for bulky stage III-IV patients who are poor surgical candidates and/or for whom there is a low likelihood of optimal cytoreduction. NACT can increase the rate of complete cytoreduction, but this advantage has not translated to an improvement in survival. Therefore, we aimed to identify factors associated with the survival of patients who received NACT followed by interval debulking surgery (IDS).

**Methods:**

A retrospective study was conducted in FIGO stage IIIC-IV epithelial ovarian cancer patients who underwent PDS or IDS in our center between January 1st, 2013, and December 31st, 2018.

**Results:**

A total of 273 cases were included, of whom 20 were lost to follow-up. Progression-free survival (PFS) and overall survival (OS) of the IDS and PDS groups were found to be similar, although the proportion of patients in stage IV and serum carbohydrate antigen 125 (CA125) levels before treatment in the IDS group were significantly higher than that in the PDS group. Body mass index (BMI), CA125 level before IDS, residual disease after surgery, and the interval between preoperative and postoperative chemotherapy were all found to be independent prognostic factors for PFS; FIGO stage, residual disease after surgery, and CA125 level before IDS were independent prognostic factors for OS. We found that PFS and OS were both significantly longer in patients with normal CA125 levels before IDS and when the interval between preoperative and postoperative chemotherapy was < 35.5 days (IDS-3 group) than for patients in the PDS group.

**Conclusions:**

The results suggested the importance of timely IDS and postoperative chemotherapy and potentially allowed the identification of patients who would benefit the most from NACT. Normal CA125 levels before IDS and an interval between preoperative and postoperative chemotherapy no longer than 5 weeks were associated with improved prognosis in advanced ovarian cancer patients.

## Background

Of the three common cancers of the female reproductive system, ovarian cancer has the highest mortality rate. In 2018, there were approximately 295,000 new cases worldwide and approximately 185,000 deaths [1]. Ovarian cancer has no specific clinical symptoms in the early stages, and effective early screening methods are lacking. Consequently, approximately 70% of patients are in the advanced stage (FIGO stage III-IV) when they first present. Currently, there are two main treatments for advanced ovarian cancer: (1) primary debulking surgery (PDS) + postoperative chemotherapy and (2) neoadjuvant chemotherapy (NACT) + interval debulking surgery (IDS) + postoperative chemotherapy. The former is the standard treatment, and the latter is for bulky stage III-IV patients who are poor surgical candidates and/or for whom there is a low likelihood of optimal cytoreduction. Optimal cytoreduction is defined as residual disease with tumors < 1 cm in size after surgery. However, maximal effort should be made to remove all visible disease (R0) regardless of whether PDS or IDS is applied because R0 is one of the most important independent prognostic factors in ovarian cancer. In contrast, the therapeutic benefit of NACT remains controversial. It is easier to achieve R0 cytoreduction with a lower incidence of complications by treating patients with NACT-IDS than by treating patients with PDS [2–6]. Nevertheless, this advantage has not translated to an improvement in survival. Three prospective randomized controlled trials concluded that the prognosis of patients treated with NACT-IDS was noninferior to that of patients treated with PDS [7–9], but a meta-analysis indicated that PDS yielded better survival than NACT-IDS [6]. There are clear recommendations concerning the treatment cycles, administration routes, and chemotherapy regimens of NACT in the National Comprehensive Cancer Network (NCCN) guidelines. However, some unanswered questions still need to be investigated and discussed, such as the optimal timing for IDS and perioperative chemotherapy. Therefore, this study aimed to identify prognostic factors associated with acceptable NACT-IDS outcomes, and we expected that controlling certain factors would improve the prognosis.

## Methods

### Study population

In this retrospective study, we analyzed the clinical data of FIGO stage IIIC-IV epithelial ovarian cancer patients who underwent PDS or IDS between January 1st, 2013, and December 31st, 2018, at the Gynecological Oncology Center of the Sichuan Cancer Hospital. Patients meeting the following inclusion criteria were enrolled in this study: (1) pathologically diagnosed in our hospital; (2) FIGO stage IIIC-IV; (3) had not received any other anti-tumor therapy other than chemotherapy and bevacizumab; and (4) had not received any poly-ADP-ribose polymerase inhibitors (PARPi) maintenance therapy.

### Treatment

After the assessment by our multidisciplinary team (MDT), NACT was considered for patients who were unlikely to be completely cytoreduced to R0 and/or who were poor candidates for surgery. R0 assessment was mainly made utilizing computed tomography (CT) or magnetic resonance imaging (MRI) before treatment. Before NACT, the diagnoses of all patients were confirmed by histological evidence on biopsy or cytopathological evidence from ascites or pleural effusion together with a ratio of carbohydrate antigen 125 (CA125) to carcinoembryonic antigen (CEA) > 25. The preferred regimen for NACT and postoperative chemotherapy was paclitaxel (135-175 mg/m^2^)/carboplatin (area under the curve (AUC) 5–6). Both PDS and IDS were performed through open laparotomy. No visible residual disease after surgery was defined as R0, residual disease after surgery < 1 cm was defined as R1, and residual disease after surgery ≥1 cm was defined as R2. The interval between preoperative and postoperative chemotherapy was measured as the duration between day 1 of the final cycle of NACT and day 1 of the first cycle of postoperative chemotherapy.

### Follow-up

All enrolled patients were followed up until May 20th, 2020, or death. Overall survival (OS) was defined from the date of pathological diagnosis to death or the end of follow-up (survivor); progression-free survival (PFS) was defined from the end of treatment to recurrence or the end of follow-up (non-relapse).

### Statistical analysis

Statistical analysis was performed using SPSS version 25.0 for Windows. Continuous variables with normal distribution are given as the means ± SD, and independent t-tests or one-way ANOVA were used for comparison; for continuous variables with non-normal distribution, the median (interquartile range (IQR)) was used for statistical description, and comparisons were performed by the Mann-Whitney U-test or Kruskal-Wallis H-test. Categorical variables are given as frequencies and percentages [n (%)], and a chi-square testing was used for comparisons between groups. Univariate and multivariate analyses of PFS and OS were conducted using Cox proportional hazards models. Kaplan-Meier curves were created for each of the clinicopathological variables to assess their associations with PFS and OS. A log-rank (Mantel-Cox) test was used to compare PFS and OS. Receiver operating characteristic (ROC) curves for the prediction model were created to obtain the AUC and analyze thresholds at which the Youden index was at its maximum. *P* < 0.05 was considered statistically significant.

## Results

### Patients’ characteristics

A total of 273 patients were included, of whom 20 (7.3%) were lost to follow-up; overall, 253 patients (92.7%) remained for the complete follow-up. The median follow-up time was 51.2 months, and the median age was 51 years (IQR: 46–60). Most patients had FIGO stage IIIC disease (*n* = 209, 82.6%), and 44 patients (17.4%) were at stage IV. There were 204 cases (80.6%) of serous carcinoma, 9 cases (3.6%) of clear cell carcinoma, 9 cases (3.6%) of mixed carcinoma, and the remaining 24 cases (9.5%) were carcinoma of unknown type. The differentiation grade of the tumors was G3 in 216 cases (85.4%). Of these patients, 133 (52.6%) were completely cytoreduced to R0, 72 (28.4%) achieved R1; thus, the optimal cytoreduction rate (R0 + R1) was 81.0%.

### Comparison of baseline characteristics between the two groups

Of the 253 patients, 90 (35.6%) were treated with PDS followed by postoperative chemotherapy, and 163 (64.4%) were treated with NACT followed by IDS and postoperative chemotherapy. The differences in FIGO stage, pathological type, Eastern Cooperative Oncology Group performance status (ECOG PS) score, serum CA125 level before treatment, and residual disease after surgery were statistically significant between these two groups (*P* < 0.05). There were more patients at stage IV in the IDS group than in the PDS group (23.9% vs. 5.6%, *P* < 0.001); the CA125 before treatment of patients in the IDS group was significantly higher (1226.00 U/ml vs. 801.20 U/ml, *P* = 0.007); the ECOG PS score was also higher in the IDS group than in the PDS group (*P* = 0.000); and the rate of achieving R0 was higher in the IDS group than in the PDS group (60.10% vs. 38.9%, *P* = 0.003). Additional information is presented in Table 1.

**Table 1:** Comparison of clinicopathological characteristics between the two groups of patients studied

	PDS group (***n*** = 90)	IDS group (***n*** = 163)	***P*** value
Age (years)	51 (46, 58)	52 (47, 60)	0.333
BMI (kg/m^2^)	22.90 (20.50, 25.11)	22.59 (20.96, 24.61)	0.801
FIGO stage			0.000
IIIC	85 (94.4%)	124 (76.1%)	
IVA	1 (1.1%)	9 (5.5%)	
IVB	4 (4.4%)	30 (18.4%)	
ECOG PS	0 (0–1)	2 (1–2)	0.000
Pathological type			0.000
Serous carcinoma	73 (81.1%)	131 (80.4%)	
Mucinous carcinoma	1 (1.1%)	2 (1.2%)	
Clear cell carcinoma	6 (6.7%)	3 (1.8%)	
Endometrioid adenocarcinoma	1 (1.1%)	2 (1.2%)	
Mixed carcinoma ^a^	7 (7.8%)	2 (1.2%)	
Carcinosarcoma	1 (1.1%)	0	
Unknown type	1 (1.1%)	23 (14.1%)	
Tumor differentiation			0.591
G1	5 (6.0%)	7 (4.8%)	
G2	0	1 (0.7%)	
G3	78 (94.0%)	138 (94.5%)	
Residual disease after surgery			0.003
R0	35 (38.9%)	98 (60.1%)	
R1	30 (33.3%)	42 (25.8%)	
R2	25 (27.8%)	23 (14.1%)	
Chemotherapy regimen			0.330
Paclitaxel/carboplatin	89 (98.9%)	160 (98.2%)	
Paclitaxel/carboplatin+ bevacizumab	0	1 (0.6%)	
Other regimens	1 (1.1%)	2 (1.2%)	
Postoperative chemotherapy cycles	7 (6, 8)	6 (5, 8)	0.051
Interval from surgery to the first cycle of postoperative chemotherapy (days)	8 (6, 10)	7 (7, 10)	0.283
CA125 before treatment (U/ml)	801.20 (359.63, 1815.25)	1226.00 (595.40, 2424.00)	0.007

^a^ Mixed carcinoma: serous carcinoma + clear cell carcinoma, serous carcinoma + endometrioid carcinoma, or endometrioid carcinoma + clear cell carcinoma

### Prognosis

Of the 253 patients who completed follow-up, 46 cases (18.3%) were stable without recurrence, 207 cases (81.8%) relapsed, and 137 cases (54.2%) died. The median PFS was 9.6 months (IQR: 7.5–11.6), and the median OS was 38.5 months (IQR: 31.5–45.5).

**Fig. 1:**
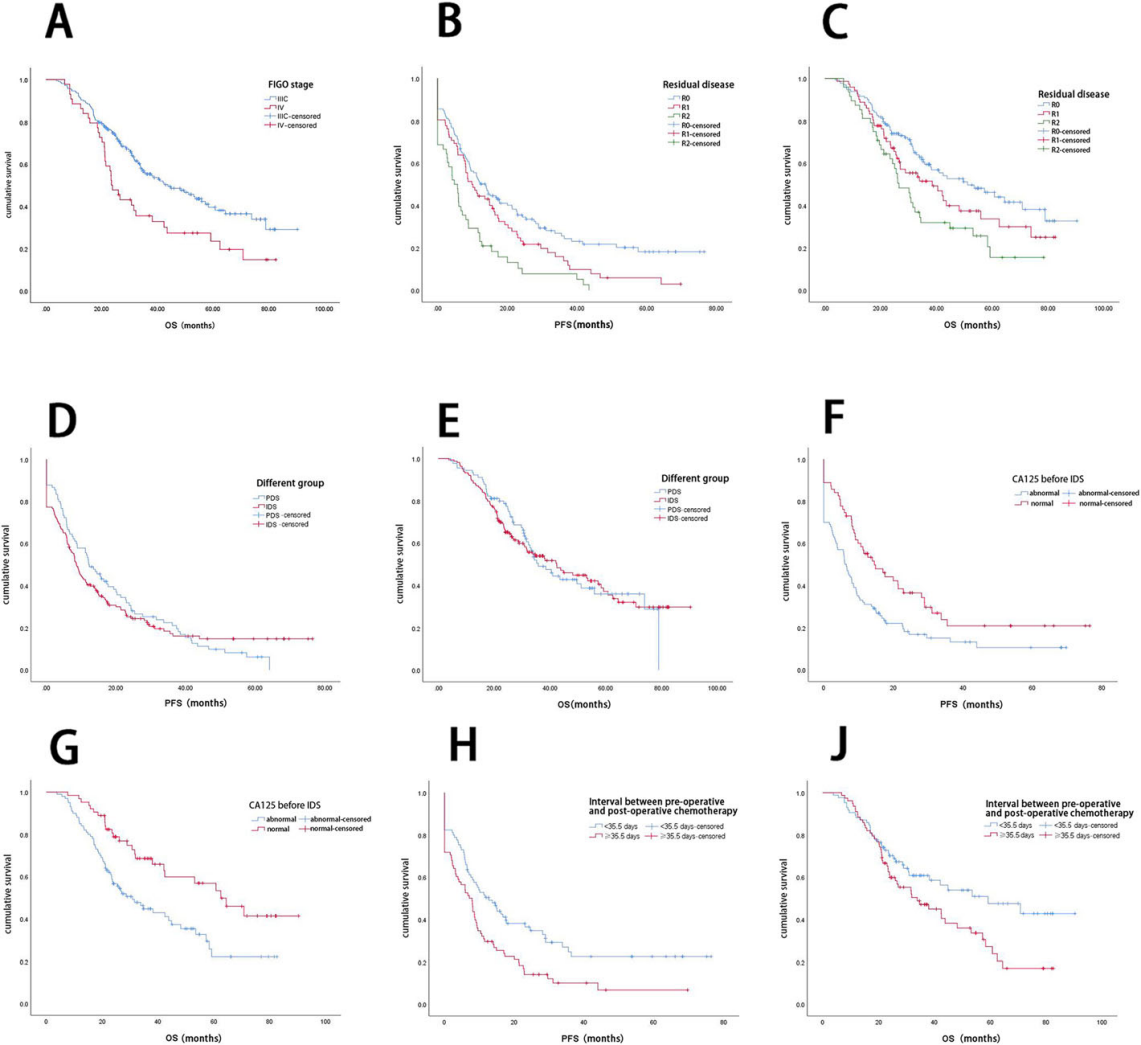
**a** OS at different FIGO stages. **b** and **c** PFS and OS according to residual disease. **d** and **e** PFS and OS of different groups. **f** and **g** PFS and OS of patients according to CA125 level before IDS. **h** and **j** PFS and OS of patients stratified by the duration of the interval between preoperative and postoperative chemotherapy

According to Kaplan-Meier analysis, the OS of stage IIIC patients was significantly longer than that of stage IV patients (median OS: 43.8 months vs. 23.5 months, *P* = 0.004) (Fig. 1a). The PFS and OS of patients with different degrees of residual disease were significantly different; the median PFS of R0, R1, and R2 patients was 13.7, 9.8, and 4.8 months, respectively (*P* = 0.000) (Fig. 1b), and the median OS was 51.1, 38.5, and 26.6 months, respectively (*P* = 0.004) (Fig. 1c). No significant differences were found between the PDS and IDS groups for PFS (12.1 vs. 8.6 months, *P* = 0.635) (Fig. 1d) or OS (35.8 vs. 42.4 months, *P* = 0.879) (Fig. 1e).

### Analysis of the IDS group

For all 163 patients in the IDS group, the median number of NACT cycles was 3 (IQR: 3–4); the median number of postoperative chemotherapy cycles was 6 (IQR: 5–8); the median interval between preoperative and postoperative chemotherapy was 35 days (IQR: 32–42); and the median interval from surgery to the first cycle of postoperative chemotherapy was 7 days (IQR: 7–10). Relapse occurred in 128 patients (78.5%), and 87 patients (53.4%) died.

Multivariate Cox regression analysis using stepwise analysis (forward: LR) indicated that body mass index (BMI), residual disease after surgery, the interval between preoperative and postoperative chemotherapy, and CA125 level before IDS were independent prognostic factors for PFS; of these factors, residual disease after surgery and CA125 level before IDS were also independent prognostic factors for OS, as was FIGO stage.

Figure 1f and g show the PFS and OS curves for patients with normal or elevated CA125 levels before IDS. Kaplan-Meier analysis indicated that the PFS of patients with normal CA125 levels before IDS was significantly longer than the PFS of patients with elevated CA125 levels (median PFS: 14.6 months vs. 6.1 months, *P* = 0.003); the OS of patients with normal CA125 levels before IDS was also significantly longer (median OS: 62.5 months vs. 30.9 months, *P* = 0.002).

### Analysis of the interval between preoperative and postoperative chemotherapy in the IDS group

**Fig. 2:**
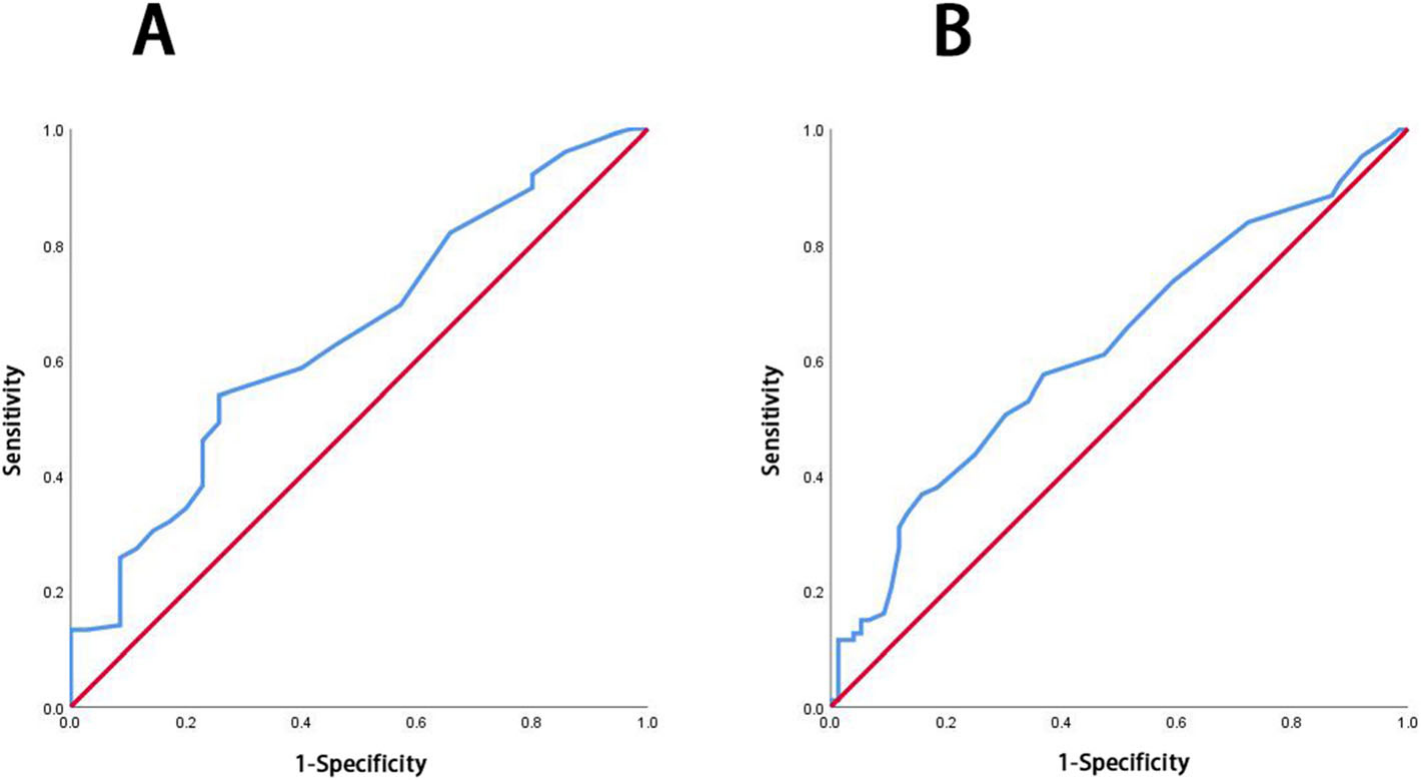
**a** ROC curve of recurrence or lack of recurrence and the interval between preoperative and postoperative chemotherapy; **b** ROC curve of mortality or survival and the interval between preoperative and postoperative chemotherapy

ROC curves for recurrence and the interval between preoperative and postoperative chemotherapy were generated (Fig. 2a). The AUC was 0.643 (95%CI: 0.542–0.743, *P* = 0.010). The threshold of the maximum Youden index was 35.5 days, the sensitivity was 53.9%, and the specificity was 74.3%. ROC curves for death and the interval between preoperative and postoperative chemotherapy (Fig. 2b) had an AUC of 0.623 (95% CI: 0.537–0.708, *P* = 0.007) with a threshold of the maximum Youden index of 35.5 days, a sensitivity of 57.5% and a specificity of 63.2%.

The log-rank (Mantel-Cox) test showed that the PFS and OS were significantly better for patients for whom the interval between preoperative and postoperative chemotherapy was < 35.5 days than for patients for whom the interval was ≥35.5 days. Thus, the median PFS was 12.7 months and 7.1 months, respectively (*P* = 0.002), and the median OS was 59.2 months and 33.8 months, respectively (*P* = 0.031). These PFS and OS curves are shown in Fig. 1h and j.

### Preliminary exploration of factors associated with improved prognosis in the IDS group

From the above analysis and the well-defined factors of FIGO stage and residual disease after surgery, we concluded that whether CA125 was normal before IDS and the interval between preoperative and postoperative chemotherapy were independent prognostic factors in the IDS group. Thus, patients who met the following three criteria were selected from the IDS group for further comparisons: (1) IDS-1 group: 63 patients with normal CA125 levels before IDS; (2) IDS-2 group: 85 patients with an interval between preoperative and postoperative chemotherapy < 35.5 days; and (3) IDS-3 group: 35 patients meeting the above two criteria at the same time. The log-rank (Mantel-Cox) test was then used to compare the IDS-1, IDS-2, and IDS-3 groups with the PDS group (see Table 2 for details). There were no significant differences in PFS and OS between the IDS-1 or IDS-2 groups and the PDS group (*P* > 0.05), but both the PFS and OS of patients in the IDS-3 group were significantly longer than those in the PDS group (*P* < 0.05).

**Table 2:** Comparison of the PDS group with different IDS groups

	n	Median PFS (95%CI) (months)	Median OS (95%CI) (months)
PDS group	90	12.1 (8.2–16.1)	35.8 (28.6–43.0)
IDS group	163	8.6 (6.9–10.3)	42.4 (29.6–55.2)
IDS-1 group	63	14.6 (7.7–21.5)	62.5 (44.5–80.4)
IDS-2 group	85	12.7 (7.4–18.0)	59.2 (30.1–88.3)
IDS-3 group	35	17.6 (0.0–36.1)^a^	NR ^ab^

^a^ When compared with the PDS group, the difference was statistically significant (*P* < 0.05)

^b^ Not reached

## Discussion

NACT is now widely accepted as an established strategy for advanced ovarian cancer patients who are not suitable for treatment with PDS. Approximately 20–30% of FIGO stage III and 40–60% of FIGO stage IV patients are currently treated with NACT-IDS in the United States [10, 11]. In the present study, 59.3% of stage IIIC and 88.6% of stage IV patients were treated with NACT-IDS. R0 cytoreduction was achieved significantly more frequently in the IDS group than in the PDS group (60.1% vs. 38.9%, respectively), this finding is consistent with the conclusions of other studies that reported the R0 rate to be 17.0–44.0% in the PDS group and 39.0–65.9% in the IDS group [2–8, 12–16]. NACT can undoubtedly improve the rate of complete cytoreduction.

In the present study, more patients in the IDS group were in stage IV, with the more significant tumor burden and worse performance status; however, in terms of survival outcomes, the PFS and OS of the IDS group patients were similar to those of the PDS group, somewhat reflecting the advantages of NACT-IDS. Regarding the baseline characteristics between the two groups, significant differences were found in the pathological types, mainly observed in the higher rate of the unknown carcinoma type in the IDS group. Because patients in the IDS group were diagnosed by tissue biopsy or cytopathology, it was more challenging to identify the pathological type due to a lack of available cells. The cancer cells were then damaged after NACT, so it was even harder to confirm the pathological type after IDS. However, serous carcinoma was the most common type of ovarian cancer, and the proportion of patients with this type was similar in the two groups; thus, it is reasonable to conclude that the two groups were similar in terms of pathological type.

In the present study, the median number of postoperative chemotherapy cycles was 6 in the IDS group, which seems to be an excessive number. In the NCCN guidelines, a minimum of 6 cycles of treatment was recommended, including at least 3 cycles of adjuvant therapy after IDS. It didn’t state the exact number of chemotherapy cycles after IDS. We typically performed 6 cycles of postoperative chemotherapy to patients with advanced (stage IIIC-IV) disease because of the high recurrence rate and no PARPi as the first-line maintenance therapy at that time.

We found that lower BMI, normal CA125 levels before IDS, R0 cytoreduction, and a shorter interval between preoperative and postoperative chemotherapy were independent factors associated with better PFS; earlier stage, R0 cytoreduction and normal CA125 levels before IDS were independent factors associated with better OS. The PFS and OS of patients with R0 cytoreduction were significantly better than those of patients with only R2 cytoreduction. The OS of stage IIIC patients was significantly better than that of stage IV patients, which was an expected result. These results are consistent with other published studies. BMI is an independent factor that affects PFS, which is shorter in patients with higher BMI. However, the relationship between obesity and prognosis in ovarian cancer is still controversial. A systematic review concluded that obesity 5 years before the diagnosis of ovarian cancer and obesity at a young age were associated with poor prognosis [17]. In contrast, another study concluded that height, weight, and BMI were not associated with prognosis in ovarian cancer [18].

NACT-IDS has been recognized as appropriate by most gynecologic oncologists and is recommended by the NCCN guidelines for strictly selected advanced ovarian cancer patients. A consensus has been reached on NACT regimens and cycles, but specific indicators of the best timing for IDS and the optimal time for chemotherapy in the perioperative period have not been clarified. There is also some controversy regarding the available data. A retrospective study completed in Denmark reported a greater risk of death for patients receiving chemotherapy > 32 days after surgery than for those patients receiving chemotherapy within 32 days, but this difference did not achieve statistical significance [19]. Another retrospective study in the United States indicated that chemotherapy delayed to > 35 days from PDS was associated with a 7% increased risk of death and concluded that starting chemotherapy between 21 and 35 days after PDS might improve survival [20]. An analysis of 191 patients with stage III-IV ovarian cancer from a prospective multicenter study revealed that the median interval from PDS to starting chemotherapy was 28 days (range 4–158). The timing of chemotherapy, FIGO stage, and residual disease after surgery were significant prognostic factors for OS in that multivariate analysis. The interval from PDS to the start of chemotherapy had no significant effect on the prognosis of patients without residual disease but was significantly related to the OS of patients with residual disease. Hence, the conclusion was that delayed chemotherapy initiation might reduce OS in patients with advanced ovarian cancer, especially after suboptimal cytoreduction [21]. However, the time of application of chemotherapy after PDS is not equivalent to the interval between preoperative and postoperative chemotherapy. Lee et al. [15] retrospectively analyzed 194 ovarian cancer patients and reported that the median interval between preoperative and postoperative chemotherapy was 42 days (range 16–178 days). They found that the PFS and OS were both worse when the interval between preoperative and postoperative chemotherapy was > 42 days. Nevertheless, they did not further analyze the prognosis-related ROC curve, threshold, and the corresponding PFS and OS but used the median interval between preoperative and postoperative chemotherapy as the cutoff point. Searle et al. [22] performed a retrospective study of 205 ovarian cancer patients who underwent IDS and reported that the interval between preoperative and postoperative chemotherapy was correlated with PFS and OS; the median interval between preoperative and postoperative chemotherapy was 63 days, and patients had worse OS when the interval between preoperative and postoperative chemotherapy was > 10 weeks (the interval was ≤10 weeks for 63.9% of the patients). In our study, the median interval between preoperative and postoperative chemotherapy was 35 days, which was much shorter than the reported intervals in the two studies mentioned above, and the interval was < 35 days in 85 cases (52.1%). We usually perform IDS approximately 3 weeks after the last cycle of NACT and start the first postoperative chemotherapy approximately 7 days after IDS if the patient recovers normally. In our experience, this is a safe and feasible approach. Furthermore, our results showed that the interval between preoperative and postoperative chemotherapy was significantly correlated with both PFS and OS; we established the threshold of 35.5 days by ROC curve analysis, such that patients with an interval between preoperative and postoperative chemotherapy of < 35.5 days had better PFS and OS, yielding an improvement in PFS and OS of 5.6 months and 25.4 month, respectively. In addition, regarding the decline in CA125 levels after NACT, a published study reported that a CA125 decrease of > 95% after NACT and CA125 level < 100 U/ml before IDS were related to better surgical outcomes and prognosis [23]. Similarly, we concluded that a normal CA125 level before IDS was associated with better OS and PFS, but in our study, a > 90% decrease in CA125 levels after NACT did not correlate significantly with optimal cytoreduction or prognosis. This discrepancy may have arisen due to different statistical analysis methods, diverse thresholds, and various variables in Cox regression analysis.

Among the above independent predictors of PFS and OS identified in the present study, normal CA125 levels before IDS and the interval between preoperative and postoperative chemotherapy (arrangement of surgery time and perioperative chemotherapy time) were the two controllable factors, so we performed a subgroup analysis on three groups of IDS patients who met these two conditions separately or at the same time. We found that the PFS and OS of all three groups were longer. However, a statistically significant difference was achieved only in patients with both a normal CA125 level before IDS and an interval between preoperative and postoperative chemotherapy < 35.5 days (the IDS-3 group); the PFS for these patients was 5.5 months longer than that of the PDS group, and the median OS of the IDS-3 group has not yet been reached. These results suggested that normal CA125 levels before IDS and an interval between preoperative and postoperative chemotherapy no longer than 5 weeks were associated with improved prognosis in advanced ovarian cancer patients. Thus, it is anticipated that under these conditions, NACT would not only increase the rate of complete cytoreduction of all visible disease (R0) but also translate into improved survival of patients with advanced ovarian cancer. However, the present investigation is limited as it is a retrospective study of a single center. Our findings need to be validated with a prospective multicenter randomized controlled study with a larger sample size.

The results of this study do not imply that we should increase the number of cycles of NACT to reduce CA125 to normal levels before IDS. Increasing the number of cycles of NACT is not a good choice because it may increase the incidence of chemotherapy resistance, and giving > 3 cycles of NACT does not change the resectability or the complete pathologic response [24]. Another study evaluating the influence of increasing NACT cycles on survival reported similar results. They found that R0 rates were similar among patients receiving 3, 4 and ≥ 5 NACT cycles (68.5, 70, and 71.4%, respectively), but patients having ≥5 NACT cycles had a poorer prognosis than those receiving 3–4 cycles [25]. Most patients treated with NACT are generally in poor condition, so the initial dose of chemotherapeutics is often limited. Active adjustment of the dose of chemotherapeutics when the patients’ general condition improves and timely modification of the chemotherapy regimen when the effect is poor may help to minimize the amount of tumor present before surgery.

NACT-IDS remains an essential method of personalized treatment for patients with advanced ovarian cancer, which increases the rate of optimal cytoreductive surgery. However, as discussed above, PFS and OS are not markedly improved. NACT-IDS is still viewed as potentially beneficial, prompting gynecologic oncologists to continue to explore improved application methods. In addition to exploring the timing of IDS and chemotherapy, more effort should be made to effectively confront the problem of chemotherapy resistance and develop new chemotherapy regimens, antiangiogenic therapies, PARPi maintenance therapies, immunotherapies, etc., which are additional promising approaches for improving the prognosis of patients with advanced ovarian cancer [26].

## Conclusions

In addition to established prognostic factors, such as FIGO stage and residual disease after surgery, the interval between preoperative and postoperative chemotherapy was found to be an independent prognostic factor for PFS; CA125 level before IDS was found to be an independent prognostic factor for both PFS and OS. Normal CA125 levels before IDS and an interval between preoperative and postoperative chemotherapy of no longer than 5 weeks were associated with improved prognosis in advanced ovarian cancer patients, which suggested the importance of timely IDS and postoperative chemotherapy and potentially allowed the identification of those patients who would benefit the most from NACT-IDS.

## Data Availability

The dataset used or analyzed in this study is available from the corresponding author upon reasonable request.

## Abbreviations

AUC: Area under the curve
BMI: Body mass index;
CA125: Carbohydrate antigen 125
CEA: Carcinoembryonic antigen
CT: Computed tomography
ECOG PS: Eastern Cooperative Oncology Group performance status
IDS: Interval debulking surgery
IQR: Interquartile range
MDT: Multidisciplinary team
MRI: Magnetic resonance imaging
NACT: Neoadjuvant chemotherapy
NCCN: National Comprehensive Cancer Network
OS: Overall survival
PARPi: Poly-ADP-ribose polymerase inhibitors
PDS: Primary debulking surgery
PFS: Progression-free survival
ROC: Receiver operating characteristic
